# Using web-sourced photographs to examine temporal patterns in sex-specific diet of a highly sexually dimorphic raptor

**DOI:** 10.1098/rsos.220779

**Published:** 2022-10-19

**Authors:** Connor T. Panter, Arjun Amar

**Affiliations:** ^1^ School of Geography, University of Nottingham, Nottingham NG7 2RD, UK; ^2^ FitzPatrick Institute of African Ornithology, DSI-NRF Centre of Excellence, University of Cape Town, Rondebosch, Cape Town, South Africa

**Keywords:** *Accipiter nisus*, citizen science, Eurasian sparrowhawk, prey selection, reversed size dimorphism, sparrowhawk

## Abstract

Traditional methods to study raptor diet are usually limited temporally, e.g. prey remains at nesting sites, and are unsuitable to examine dietary changes throughout the year. Using web-sourced photography, we explore temporal patterns in prey size and key prey species between sexes of the sexually dimorphic Eurasian sparrowhawk (*Accipiter nisus*) throughout the United Kingdom. We examined 666 photographs of sparrowhawk on prey and identified the prey species involved, together with sparrowhawk sex. Changes in prey size and proportions of key prey species over time (seasonally and monthly) were explored for each sex. Prey weight was substantially higher for females than males. However, on average, prey size for both sexes declined during the summer period (May–June) being the lowest in June, which is the main nestling-rearing month for both sparrowhawks and their prey. Compared with summer, rock doves (*Columba livia*) were more important prey for female sparrowhawk in winter. Whereas, for males, Eurasian blackbirds (*Turdus merula*) were more important in spring compared with autumn. Web-sourced photography can overcome several limitations of previous methods used to study raptor diet including the ability to quantify diet between the sexes throughout the entire year, however, may also introduce a prey-size bias toward larger prey items.

## 1. Introduction

Knowledge of a species' diet is essential to fully understand its ecology and resource requirements [1]. Such information provides the foundation for understanding inter- and intra-specific interactions [2,3], which structure ecological communities and control energy flow and nutrients through food webs [4]. Understanding dietary requirements and whether these vary over time may also be important for effective conservation planning [5].

In many bird species differences in diet exist between the sexes. This may arise via sexual differences in habitat selection [6,7], foraging behaviour [8,9], food intake patterns [10,11] or digestion [9], and may be most pronounced in birds that show strong sexual size dimorphism [12], such as raptors [13].

Raptors generally show sexual dimorphism (SD), with females being the larger sex [12,14,15]. The degree of SD in raptors is strongly linked to diet with species feeding on the most agile prey, for example, bird-eating raptors, showing the greatest size differences between the sexes [13,16]. One explanation proposed for SD in raptors is the ‘intersexual competition’ hypothesis [13,15]. This hypothesis proposes that the sexes' different sizes evolved as a means to exploit different prey size niches to minimize intersexual competition for food resources during the nestling period when both sexes are foraging in the same territory to provide food for their nestlings [17,18]. Sexual differences in the diet of raptors have frequently been observed, with females often taking large prey items [19,20,21].

Methods used to study raptor diet, such as analysis of prey remains, pellets, hide watches [2,22–24] and more recently nest cameras [25,26], may be limited in their ability to distinguish between the diet of different sexes or ages or may be limited to specific periods of time, particularly the breeding season, with the nest site being the focus of data collection. Consequently, studies of raptor diet tend to focus on the breeding season, with far less focus outside of this period [27,28], and thus very rarely explore temporal differences in diet throughout the year [29,30].

Several approaches have sought to overcome the limitations of conventional methods to study raptor diet over time, for example, radiotelemetry has been used to study raptor diet throughout the year in Cooper's hawks (*Accipiter cooperii*) [31,32] and northern goshawks (*A. gentilis*) [33,34]. Stable isotope analysis has been used to explore seasonal diet in white-tailed eagles (*Haliaeetus albicilla*) [35], and faecal analyses have been used to explore the diet of Lanyu scops owls (*Otus elegans*) over time [18]. However, such methods can be costly, labour intensive or may require high degree of expertise (e.g. specialist laboratory skills). A study by Bujoczek & Ciach [30], examined diet differences in Eurasian sparrowhawk (*Accipiter nisus*) by examining prey remains throughout the year within a main hunting area but were unable to distinguish prey remains attributed to sparrowhawk sex.

The use of web-sourced photographs to study raptor diet is a promising new method that has the potential to overcome several limitations of previous approaches, such as allowing diet to be examined throughout the year, and between sexes and ages, or over wider geographical areas. For example, Naude *et al.* [36] used web-sourced photographs to explore geographical variations in martial eagle (*Polemaetus bellicosus*) diet across sub-Saharan Africa and to explore diet differences between adults and non-adults. Similarly, Panter & Amar [21] used web-sourced photographs to explore sex- and age-related diet differences in Eurasian sparrowhawks across the UK. Berryman & Kirwan [37] looked at the diet of tiny hawks (*Accipiter superciliosus*) using web-sourced photographs, and a more recent study used web-sourced imagery to contribute to conservation and law enforcement policies [38]. Except for human time, analysing web-sourced photographs requires relatively minimal skill other than knowledge of visual characteristics of predators (e.g. sex and age) and their prey species.

In this study, we expand on Panter & Amar [21] and explore the diet of the Eurasian sparrowhawk (hereafter ‘sparrowhawk’) over time. We use web-sourced photographs to examine how the species' diet changes across the entire year between the sexes. Sparrowhawks display extreme size dimorphism, being arguably one of the most sexually dimorphic raptors, with females being nearly twice the size of the male (adult female body mass: 258–325 g vs adult male: 143–155 g [39]). We explore seasonal differences in the size of prey of male and female sparrowhawks throughout the year, and for several commonly occurring prey species, we examine how these key prey species vary seasonally in importance within sparrowhawk diet for the different sexes.

## 2. Methods

### 2.1. Web-sourced data

**Figure 1.**
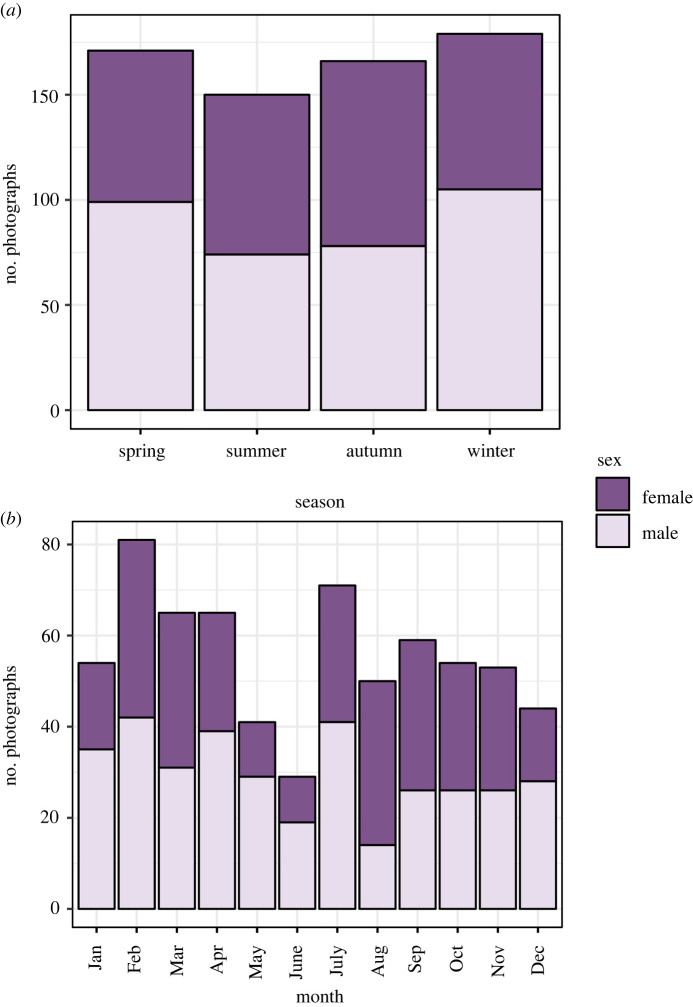
Sample sizes for photographs of adult male (*N* = 356) and female (*N* = 310) Eurasian sparrowhawk (*Accipiter nisus*) on prey in the United Kingdom (*a*) across seasons and (*b*) by month.

Photographs of sparrowhawks on prey within the United Kingdom (UK) were collated between 24 July and 16 August 2019, from various web sources [21]. Initially, we used the web-application MORPHIC ([40]; http://morphs.io/) which uses specified search terms to retrieve photographs from the search engine Google Images (full methodological details are provided in [21]), together with manual searches on Facebook (www.facebook.com), Twitter (www.twitter.com) and BirdGuides (www.birdguides.com). Additional searches of these same sites were also then carried out between 20 January and 8 February 2021 to supplement the original data from Panter & Amar [21]. Additionally, we posted a public appeal via Twitter on 8 February 2021, requesting extra photographs including those specifically taken in May, June and December for which we had the lowest sample sizes (figure 1), but also received and accepted photos taken at other times of the year.

For photographs that contained a wild sparrowhawk with its prey, the following data were collected: (i) sparrowhawk age (juvenile < 2nd calendar year > adults), (ii) sex (male or female), (iii) prey species (identified to the lowest taxonomic level possible), and (iv) observation date. The ages and sexes of the sparrowhawks were further confirmed by having multiple experienced ornithologists view each photo. For this current analysis, only data for adult birds were used due to the comparatively low sample sizes, between the sexes, for juvenile sparrowhawks. For further methodological details see Panter & Amar [21]. Using the month of observation for each photo we defined a four-season variable as follows: ‘spring’ (March, April and May), ‘summer’ (June, July and August), ‘autumn’ (September, October and November) and ‘winter’ (December, January and February). Sparrowhawks are a single brooding species which, in the UK, typically lay between April and May (mean 11 May) [41]. Incubation lasts *ca* 33 days and the nestling period until fledging lasts *ca* 30 days [41]. Thus, the main nestling period would be in June and July, and our ‘summer’ season would have captured the main period where breeding birds have nestlings or larger dependent young.

### 2.2. Prey weight

For each prey item we determined a prey weight (table 1; [41]), and allocated prey items to the following three size categories: small (≤35 g), medium (≥36g to ≤120 g) or large (≥121 g). Some prey were identified as Columbidae spp. but not to species level, these items were given the average weight of all identified Columbidae prey in the diet. We grouped all *Columba livia* (e.g. rock doves, feral pigeons and white doves) under the prey item ‘rock dove’. Other prey unidentified to species level were categorized into one of three size categories: ‘unidentified small bird sp.’, ‘unidentified medium bird sp.’ and ‘unidentified large bird sp.’. We calculated mean prey weights for unidentified prey by taking the mean value for all items within each respective size class. It was not possible to collect data on prey age due to difficulty in ascribing age classes to prey caught from a single photograph.

**Table 1.** Mean prey weight estimates for female (*N* = 310) and male (*N* = 356) Eurasian sparrowhawk (*Accipiter nisus*) throughout the United Kingdom, by season and month. Estimates were from a general linear model fitted with sex × season, or sex × month. Seasonal and monthly comparisons between the sexes were calculated from EMMEANs contrasts. CI = confidence intervals. Significant values in italic.

	female		male		pairwise comparisons	
	mean prey weight (g)	95% CI range	mean prey weight (g)	95% CI range	*t*	*p*
season						
*spring*	*300.2*	*(261.3–339)*	*127.7*	*(100.7–154.7)*	*7.337*	*<0.0001*
*summer*	*258.2*	*(220.4–296)*	*88.4*	*(57.2–119.6)*	*6.850*	*<0.0001*
*autumn*	*315.9*	*(280.8–351)*	*125.0*	*(94.6–155.4)*	*8.089*	*<0.0001*
*winter*	*321.6*	*(283.3–359.9)*	*123.0*	*(96.8–149.2)*	*8.622*	*<0.0001*
month						
*Jan*	*289.0*	*(214.8–363.3)*	*118.3*	*(73.2–163.5)*	*4.000*	*0.015*
*Feb*	*333.8*	*(282.0–385.6)*	*157.1*	*(115.9–198.3)*	*5.306*	*<0.0001*
*Mar*	*302.7*	*(247.2–358.2)*	*156.3*	*(108.3–204.3)*	*3.938*	*0.019*
*Apr*	*333.2*	*(269.7–396.6)*	*125.7*	*(82.9–168.5)*	*5.472*	*<0.0001*
May	221.3	(127.9–314.7)	99.8	(50.2–149.4)	2.365	0.773
Jun	126.2	(23.9–228.5)	66.2	(5.0–127.5)	1.026	1.000
*Jul*	*232.2*	*(173.2–219.3)*	*79.4*	*(37.7–121.1)*	*4.248*	*0.006*
Aug	316.4	(262.5–370.3)	144.7	(73.3–216.1)	3.640	0.052
*Sep*	*354.4*	*(298.1–410.8)*	*107.3*	*(54.9–159.7)*	*6.293*	*<0.0001*
*Oct*	*301.4*	*(240.2–362.5)*	*129.1*	*(76.7–181.4)*	*4.225*	*0.006*
Nov	283.9	(221.6–346.2)	138.6	(86.3–191)	3.530	0.074
*Dec*	*330.6*	*(249.7–411.5)*	*77.7*	*(27.3–128.2)*	*5.388*	*<0.0001*

### 2.3. Statistical analysis

All statistical analyses were performed in R v. 3.6.3 [42]. We explored differences in prey weight between the sexes across seasons (spring, summer, autumn and winter) using a linear model (LM) with estimated weight for each prey item as the response variable and sex, season and their interaction (sex × season) fitted as explanatory variables. We then repeated this analysis but substituted season for month and fitted sex, month and their interaction (sex × month) as explanatory variables. These analyses explored whether any changes across time were similar between the sexes and whether differences in prey weight between the sexes occurred during the different parts of the year.

We then repeated the LMs separately for each sex to explore within-sex diet differences over time. Mean prey weight was fitted as the response variable and season fitted as the explanatory variable. These models were then repeated with month fitted as the explanatory variable, to explore monthly changes in the sparrowhawk prey weight for each sex.

To explore differences in prey size class (small, medium and large) between the sexes over time, we used a pairwise multimodal log-linear model implemented with the ‘NNET’ package [43]. Proportional prey size category was fitted as the response variable, and sex, season and their interaction (sex × season) fitted as explanatory variables. We also explored changes in prey size category by month, replacing season with month and fitting sex, month and their interaction (sex × month) as additional explanatory variables. These models were then repeated to explore changes in prey size class within sexes over time, with prey size class fitted as the response variables and separate models with season and month fitted as explanatory variables.

Lastly, we explored how the relative proportion of key prey species of the different sexes varied across seasons. For this, we selected only those identified prey species that represented greater than or equal to 5% of sparrowhawk diet for each sex across the year, hereafter referred to as ‘key prey species’. For each of these key prey species, we created a binary response variable, scoring each photograph as either 1 or 0 depending on whether the photo was of that prey species (1) or not (0). We then fitted a series of generalized linear models (GLMs) for each sex and each key prey species, with the binary presence/absence prey variable fitted as the response variable and season fitted as the explanatory variable. We performed *post hoc* pairwise comparisons to examine the proportion of that prey type between seasons. Due to the relatively small sample sizes, we did not repeat these analyses by month. These GLMs were run using binomial error distributions with ‘logit’ link functions. Means (±95% confidence intervals) for each sex in each season/month were generated using the ‘EMMEANS’ package [44].

## 3. Results

In total, we obtained 666 photographs of adult sparrowhawks on prey items in the UK, with similar numbers for each sex (356 males; 310 females). The mean number of photographs in each season for females was 76 ± 6 (±s.d.) (range: 72–88) and for males was 89 ± 13 (range: 74–105) (figure 1*a*). The mean number of monthly photographs was 26 ± 9 for females (range: 10–39) and 30 ± 8 for males (range: 14–42) (figure 1*b*). There was a decline in the number of photos in May and June, particularly for females.

### 3.1. Changes in prey weight over time

**Figure 2.**
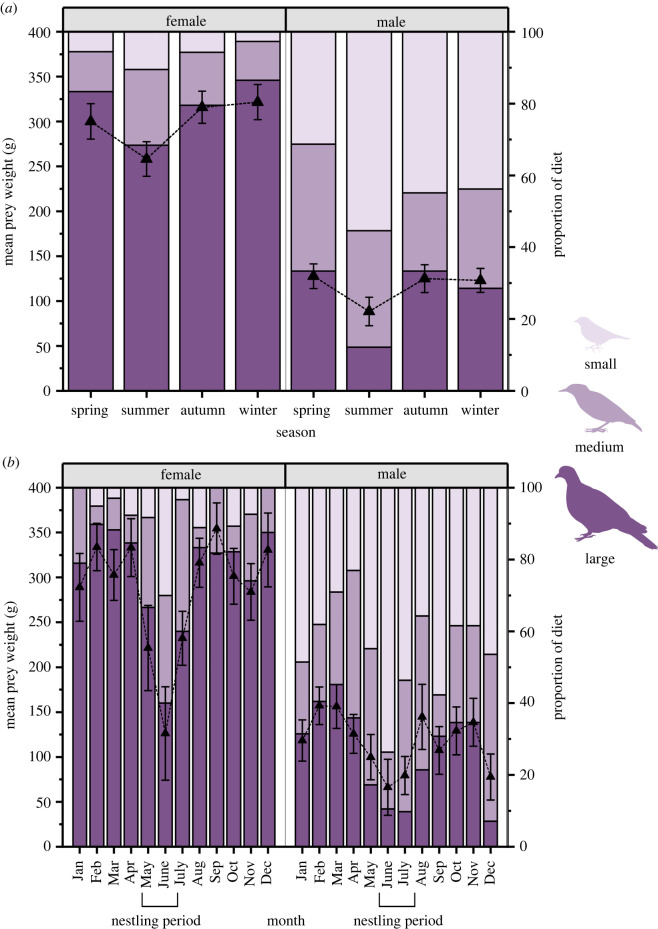
Prey weights (g) and proportion of different prey size classes (small, medium and large) within the diet of adult male and female Eurasian sparrowhawk (*Accipiter nisus*) in the United Kingdom (*a*) across seasons and (*b*) by month. Error bars = standard error.

Across all seasons, female sparrowhawks preyed on items that were significantly heavier than those taken by the male (*F*_1,661_ = 240.22, *p* = < 0.0001; table 1; figure 2*a*). Prey weight differences across the year were similar between the sexes, with no significant interactions in mean prey weight between the sexes and either season (sex × season: *F*_3,658_ = 0.35, *p* = 0.78) or month (sex × month: *F*_11,642_ = 1.21, *p* = 0.27). We found a significant seasonal effect (*F*_3,661_ = 3.57, *p* < 0.05) with prey weights being significantly lighter in summer compared with both winter and autumn (figure 2*a*; table 2). For most of the year, prey weights between the two sexes differed significantly. However, in May, June, August and November, differences in prey weight between the sexes were non-significant (table 1; figure 2*b*).

**Table 2.** Differences in the proportional probability of large prey items (≥121 g) in the diet of Eurasian sparrowhawk (*Accipiter nisus*) by season and month throughout the United Kingdom. Pairwise comparisons show differences between the sexes (female–male). Numerator degrees of freedom = 16 (seasons) and 48 (months), CI = confidence intervals. Significant values in italic.

	female		male		pairwise comparisons	
	probability (large)	95% CI range	probability (large)	95% CI range	*t*	*p*
season						
* spring*	*0.83*	*(0.73–0.93)*	*0.33*	*(0.22–0.44)*	*7.739*	*<0.0001*
* summer*	*0.68*	*(0.56–0.81)*	*0.12*	*(0.03–0.21)*	*8.592*	*<0.0001*
* autumn*	*0.80*	*(0.70–0.89)*	*0.33*	*(0.21–0.46)*	*6.742*	*<0.001*
* winter*	*0.86*	*(0.77–0.96)*	*0.29*	*(0.18–0.39)*	*9.757*	*<0.0001*
month						
* Jan*	*0.79*	*(0.60–0.98)*	*0.31*	*(0.15–0.48)*	*3.892*	*0.045*
* Feb*	*0.90*	*(0.80–1.00)*	*0.40*	*(0.25–0.56)*	*5.475*	*<0.001*
* Mar*	*0.88*	*(0.77–1.00)*	*0.45*	*(0.27–0.64)*	*4.098*	*0.025*
* Apr*	*0.85*	*(0.70–0.99)*	*0.36*	*(0.20–0.52)*	*4.664*	*<0.01*
May	0.67	(0.39–0.95)	0.17	(0.03–0.32)	3.228	0.217
Jun	0.40	(0.08–0.72)	0.11	(−0.04–0.25)	1.732	0.983
* Jul*	*0.60*	*(0.42–0.78)*	*0.10*	*(0–0.19)*	*4.988*	*<0.01*
* Aug*	*0.83*	*(0.71–0.96)*	*0.21*	*(−0.01–0.44)*	*4.911*	*<0.01*
* Sep*	*0.82*	*(0.68–0.96)*	*0.31*	*(0.12–0.49)*	*4.529*	*<0.01*
* Oct*	*0.82*	*(0.67–0.97)*	*0.35*	*(0.15–0.54)*	*4.024*	*0.031*
Nov	0.74	(0.57–0.91)	0.35	(0.15–0.54)	3.137	0.260
* Dec*	*0.88*	*(0.70–1.05)*	*0.07*	*(−0.03–0.17)*	*8.374*	*<0.0001*

For both males and females, prey weights appeared to decline in the summer season (figure 2*a*), but this trend was marginally non-significant for females (*F*_3,306_ = 2.25, *p* = 0.081; figure 2*a*), and was non-significant for males (*F*_3,352_ = 1.44, *p* = 0.230; figure 2*a*). However, for females, prey weights differed between months (*F*_11,298_ = 2.41, *p* < 0.01). For males, prey weights did not differ between months (*F*_11,344_ = 1.50, *p* = 0.12) despite a notable decline during the nestling period (figure 2*b*).

### 3.2. Changes in prey size classes over time

Across all seasons, the proportion of large prey items within the female diet was significantly higher than the male diet (table 2; figure 2*a*), with a similar pattern seen across months, excluding May, June and November, where no significant differences were found (table 2; figure 2*b*). In spring, the proportion of medium prey items in the diet of males was significantly higher than for females (table 3). There were no significant differences in the proportion of medium-sized prey items between sexes across any month (table 2; figure 2*b*). Small prey items were significantly higher in proportion in the male diet, across all seasons than the female diet (table 4). However, on a month-by-month basis, these significant differences were only present in January, February, July, September and December (table 4; figure 2*b*).

**Table 3.** Differences in the probability of medium prey items (≥36 g to ≤120 g) in the diet of Eurasian sparrowhawk (*Accipiter nisus*) by season and month throughout the United Kingdom. Pairwise comparisons show differences between the sexes (female – male). Degrees of freedom = 16 (seasons) and 48 (months), CI = confidence intervals. Significant values in italic.

	female		male		pairwise comparisons	
	probability (medium)	95% CI range	probability (medium)	95% CI range	*t*	*p*
season						
*spring*	*0.11*	*(0.03–0.20)*	*0.35*	*(0.24–0.46)*	*−3.996*	*0.018*
summer	0.21	(0.10–0.32)	0.32	(0.20–0.45)	−1.585	0.751
autumn	0.15	(0.06–0.23)	0.22	(0.11–0.33)	−1.167	0.930
winter	0.11	(0.02–0.19)	0.28	(0.18–0.38)	−2.968	0.122
month						
Jan	0.21	(0.02–0.40)	0.20	(0.06–0.34)	0.090	1.000
Feb	0.05	(−0.02–0.12)	0.21	(0.08–0.34)	−2.248	0.826
Mar	0.09	(−0.01–0.19)	0.26	(0.10–0.42)	−1.837	0.968
Apr	0.08	(−0.03–0.18)	0.41	(0.25–0.57)	−3.526	0.113
May	0.25	(−0.01–0.51)	0.38	(0.19–0.57)	−0.839	1.000
Jun	0.30	(0–0.60)	0.16	(−0.01–0.33)	0.849	1.000
Jul	0.37	(0.19–0.55)	0.37	(0.21–0.52)	0.007	1.000
Aug	0.06	(−0.02–0.13)	0.43	(0.16–0.70)	−2.709	0.523
Sep	0.18	(0.04–0.32)	0.12	(−0.01–0.24)	0.723	1.000
Oct	0.07	(−0.03–0.17)	0.27	(0.09–0.45)	−1.984	0.935
Nov	0.19	(0.03–0.34)	0.27	(0.09–0.45)	−0.732	1.000
Dec	0.12	(−0.05–0.30)	0.46	(0.27–0.66)	−2.705	0.526

**Table 4.** Differences in the probability of small prey items (≤35 g) in the diet of Eurasian sparrowhawk (*Accipiter nisus*) by season and month throughout the United Kingdom. Pairwise comparisons show differences between the sexes (female – male). Degrees of freedom = 16 (seasons) and 48 (months), CI = confidence intervals. Significant values in italic.

	female		male		pairwise comparisons	
	probability (small)	95% CI range	probability (small)	95% CI range	*t*	*p*
season						
*spring*	*0.06*	*(−0.01–0.12)*	*0.31*	*(0.21–0.42)*	*−4.782*	*0.003*
*summer*	*0.11*	*(0.02–0.19)*	*0.55*	*(0.42–0.69)*	*−6.632*	*<0.001*
*autumn*	*0.06*	*(0–0.11)*	*0.45*	*(0.32–0.58)*	*−6.373*	*<0.001*
*winter*	*0.03*	*(−0.02–0.07)*	*0.44*	*(0.33–0.55)*	*−7.911*	*<0.0001*
month						
*Jan*	*0.00*	*(<0.1–<0.1)*	*0.49*	*(0.31–0.66)*	*−5.749*	*<0.001*
*Feb*	*0.05*	*(−0.02–0.12)*	*0.38*	*(0.23–0.54)*	*−3.979*	*0.035*
Mar	0.03	(−0.03–0.09)	0.29	(0.12–0.46)	−3.015	0.325
Apr	0.08	(−0.03–0.18)	0.23	(0.09–0.37)	−1.802	0.974
May	0.08	(−0.08–0.25)	0.45	(0.26–0.64)	−2.990	0.340
Jun	0.30	(0.0–0.60)	0.74	(0.53–0.95)	−2.472	0.688
*Jul*	*0.03*	*(−0.03–0.10)*	*0.54*	*(0.38–0.70)*	*−5.956*	*<0.0001*
Aug	0.11	(0–0.22)	0.36	(0.09–0.62)	−1.778	0.977
*Sep*	*0.00*	*(<0.1–<0.1)*	*0.58*	*(0.38–0.78)*	*−5.954*	*<0.0001*
Oct	0.11	(−0.01–0.23)	0.38	(0.19–0.58)	−2.479	0.684
Nov	0.07	(−0.03–0.18)	0.38	(0.19–0.58)	−2.877	0.409
*Dec*	*0.00*	*(<0.1–<0.1)*	*0.46*	*(0.27–0.66)*	*−4.926*	*0.002*

Across seasons there was no significant difference in proportional prey size classes within the female diet (*X*^2^ = 8.998, d.f. = 6, *p* = 0.173; figure 2*a*); however, proportional prey size classes differed significantly when analysing female diet by month (*X*^2^ = 41,138, d.f. = 22, *p* < 0.01; figure 2*b*). For males, there was a significant difference in proportional prey size classes across seasons (*X*^2^ = 19.233, d.f. = 6, *p* < 0.01; figure 2*a*), which was also present when analysing male diet by month (*X*^2^ = 49.755, d.f. = 22, *p* < 0.001; figure 2*b*).

### 3.3. Changes in key prey over time

The key prey species (those comprising greater than or equal to 5% of identified prey species for each sex) for females included five species (common wood pigeon (*Columba palumbus*; 29% of diet), Eurasian collared dove (*Streptopelia decaocto*; 17.4%), rock dove (*C. livia*; 16.4%), common starling (*Sturnus vulgaris*; 6.7%) and Eurasian blackbird (*Turdus merula*; 5.1%)), and for males included six species (common starling (14%), house sparrow (*Passer domesticus*; 12.6%), Eurasian blackbird (8.4%), common wood pigeon (7.8%), Eurasian collared dove (7.5%) and European goldfinch (*Carduelis carduelis*; 5.3%)).

**Figure 3.**
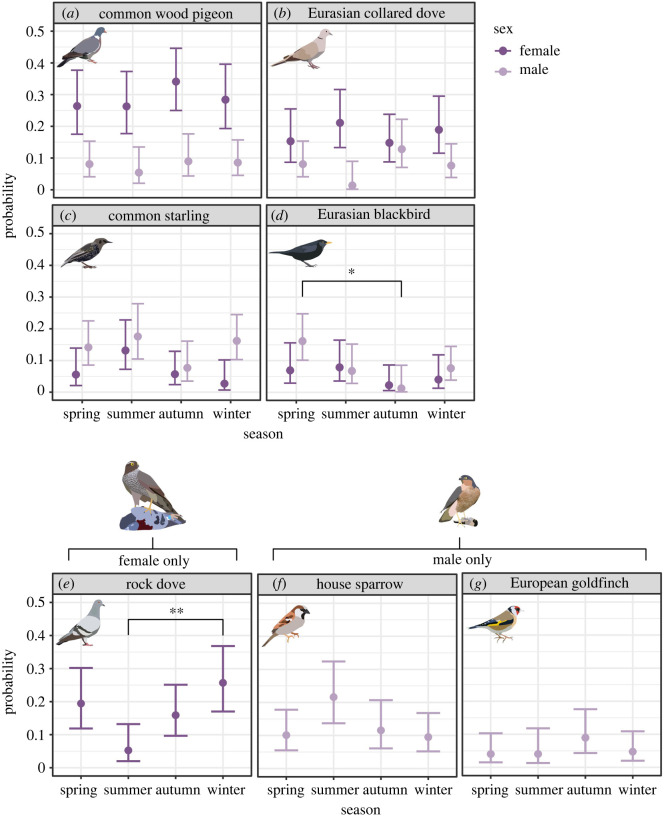
Seasonal patterns in probabilities of key prey species in the diet of adult Eurasian sparrowhawk (*Accipiter nisus*) in the United Kingdom: (*a*) common wood pigeon (*Columba palumbus*), (*b*) Eurasian collared dove (*Streptopelia decaocto*), (*c*) common starling (*Sturnus vulgaris*), (*d*) Eurasian blackbird (*Turdus merula*), (*e*) rock dove (*Columba livia*), (*f*) house sparrow (*Passer domesticus*) and (*g*) European goldfinch (*Carduelis carduelis*). Statistical significance thresholds: ‘**’ = *p* < 0.01 and ‘*’ = *p* < 0.05. Error bars = 95% confidence intervals.

Overall, there was a significant seasonal effect for rock doves within the female sparrowhawk diet (*X*^2^_3,306_ = 13.49, *p* < 0.01; figure 3). There were also significant seasonal effects for Eurasian collared doves (*X*^2^_3,352_ = 8.68, *p* < 0.05; figure 3) and Eurasian blackbirds in the male diet (*X*^2^_3,352_ = 14.37, *p* < 0.01; figure 3). The probability of rock doves in the female diet was substantially and significantly lower in summer (5%) compared with winter (20%), (*z* = −3.15, *p* < 0.01; figure 3; table 3) and marginally non-significant when compared with spring (15%) (*z* = 2.47, *p* = 0.06; figure 3; table 3). For male sparrowhawks, the probability of Eurasian collared doves in the summer diet was lower (*ca* 1%) compared with other seasons: spring (8%), autumn (13%) and winter (7%); however, within-season comparisons were non-significant (figure 3). The probability of Eurasian blackbirds in the male diet was substantially higher in spring (16%) compared with autumn (1%), a difference which was significant (*z* = 2.59, *p* < 0.05; figure 3; table 3). Although non-significant, a similar pattern was also observed for females with the species being least important during the autumn (figure 3; table 3).

## 4. Discussion

In this study, we demonstrate, for the first time, how web-sourced photographs can be used to explore temporal patterns in sex-specific diet of male and female Eurasian sparrowhawk throughout the entire year. Across all seasons females consistently hunted heavier prey than males. For both sexes, mean prey weights declined in a similar manner during the summer period, this was driven by a reduction in large prey items and an increase in both medium, and particularly, small-sized prey items during this period. Examining monthly sex differences in prey weights and size classes, we found that during May and June, there was no significant difference in the mean prey weight or the proportion of large prey items between the sexes. Thus, during these months, males and females were bringing in similar-sized items. The reduction in prey size, and the decrease in large prey items for females was particularly marked in June, which is when most breeding pairs will have chicks [45]. Our findings support those by Storer [46] who analysed stomach contents of sharp-shinned hawks (*Accipiter striatus*), Cooper's hawks (*A. cooperii*) and northern goshawks (*A. gentilis*) revealing sexual differences in diet for all three species outside of the breeding season.

Our finding of reduced prey weight during the breeding season supports those from Bujoczek & Ciach [30]; they examined sparrowhawk prey size across the breeding season and found that the proportion of small- and medium-sized prey increased, relative to large prey, during the nestling period. During the breeding season, it has been shown that sparrowhawk time their breeding period to coincide with the emergence of fledgling passerines [29], which may explain why prey weight declined for both sexes during the breeding season. Our findings are also consistent with Millsap *et al*.'s [31] and Kennedy & Johnson's [47] studies of Cooper's hawk that found similar-sized prey between the sexes during the breeding season. Similarly, Boal & Mannan [48] reported no differences in mean prey mass, between the sexes, of northern goshawk during the breeding season in Arizona, USA. By contrast, some other studies of *Accipiters* have found sex-differences in prey size during the breeding season. For example, Snyder & Wiley [17] reported significant sex differences in the weight of prey delivered to the nest in both sharp-shinned hawks and Cooper's hawks during the breeding season.

Our results of similar-sized prey items being delivered between the sexes during the nestling period, run counter to those predicted based on the ‘intersexual competition’ hypothesis proposed for the evolution of sized-based sexual dimorphism in raptors [13,49]. This hypothesis proposes that the size difference between the two sexes evolved as a means to exploit different prey size niches in order to minimize intersexual competition for food resources during the nestling period when both sexes are foraging in the same territory to provide food for their nestlings [17,18]. This hypothesis would therefore predict that differences in prey sizes between the sexes should be particularly pronounced during the nestling period, which we did not find.

Using our novel approach, we were also able to examine how the importance of the key prey species changed over time. From these analyses, we found that two prey species showed significant changes in the proportions of the sparrowhawk diet over seasons. For females, rock doves (principally made up of feral pigeons in our study) declined significantly in the female summer diet when compared with winter. This matches with a decline in all prey within the large size class which occurred during this time but was not seen in the proportion of the other large key prey species also within the large size class (e.g. common wood pigeons or Eurasian collared doves), which showed no such major reduction in their importance in summer. Thus, the pattern in decreasing prey size is probably driven by the reduction in importance of this one large (main) prey species during the summer period. One explanation for the reduction in the importance of this prey item at this time might be that rock doves represent particularly dangerous prey items for females to tackle, and thus they avoid this risky behaviour during this period when they have chicks, since any injury at the time would compromise that year's entire reproductive output.

During spring, Eurasian blackbirds increased significantly in the male diet when compared with autumn. These differences may be due to different seasonal behaviours, increasing or decreasing their vulnerability to capture at these different times. For example, increased vulnerabilities may occur in spring, due to territorial behaviour or having to feed in the open more frequently, whereas food abundance for Eurasian blackbirds may be higher in autumn, e.g. ripening and increased abundance of fruit [50], and this may decrease vulnerability to attacks by sparrowhawks. Dietary changes between seasons and between sexes have been reported in other raptors. For example, Catry *et al.* [51] found changes in diet composition before laying, which they attributed may be due to the different energetic requirements of male and female lesser kestrels (*Falco naumanni*); and Lee & Severinghaus [18] reported sex differences in the proportions of prey in the Lanyu scops owls (*Otus elegans*) between the breeding and non-breeding seasons.

While our method of studying diet using web-sourced photographs overcomes many of the limitations of other methods, it is not without its own biases or weaknesses. For example, web-sourced photography does not allow for prey to be attributed to the individual or pair level. This constrains our ability to explore how diet may affect individual- or pair-level differences (e.g. [21,26,52–54]), or to explore which environmental factors may influence diet [55–58]. Furthermore, our approach is probably biased toward large prey items, as these are more visible to observers and more likely to be consumed at the location of the kill. They may also take longer to consume, and previously we have estimated that in relation to small prey items, our method may over-represent large and medium prey items by 25% and 72%, respectively (see [21] for details). However, other methods of studying raptor diet, such as analysis of prey remains, are also known to be biased toward larger prey and mammals over birds, as such items are more likely to be detected [21,22,59].

The prey species we identified included those commonly associated with urban areas, but this was not exclusively the case, and prey items also included species associated with more rural or coastal environments, e.g. northern lapwing (*Vanellus vanellus*), grey partridge (*Perdix perdix*) and common ringed plover (*Charadrius hiaticula*). The number of photographs obtained for each sex was relatively consistent between months, except for females in May (*N* = 12) and June (*N* = 10), which may be due to the female incubating or brooding young during these months, and therefore hunting less [45].

Our approach of using web-sourced photographs can be applied to any well-sampled species, and advances in the collation and storage of natural history photographs, i.e. on iNaturalist (https://www.inaturalist.org/), Macauley Library (https://www.macaulaylibrary.org/) and BirdGuides (https://www.birdguides.com/), have increased accessibility for citizen science data to be used in ecological research. Nowadays, photographs are often geo-referenced and include time stamps which allow for research questions focusing on spatial and temporal aspects to be explored.

Our method allowed for sex-specific patterns in the diet of a raptor to be explored throughout the entire year. Most other dietary methods, such as the analysis of pellet and prey remains, do not allow diet to be explored throughout the entire year and are often biased toward the breeding season [29,30]. One of the few exceptions is the use of staple isotope analyses or radiotelemetry/GPS trackers coupled with activity sensors [33–35]; however, both methods have other limitations, either in terms of the likely sample sizes (number of different individuals in the case of radiotelemetry) or in terms of technical expertise (in the case of stable isotopes). In addition, most dietary studies are confined geographically and tend to focus on the diet of individual birds or multiple breeding pairs [60]. Our method allows for the examination of population-level diet patterns conducted at a national scale. The use of web-sourced photography is an accessible method to study diet and may be especially useful for researchers without access to funds and/or expertise in other more specialist methods. Processing and examining the photographs took around one month, and other than time involved almost no cost. The method requires minimal skill other than knowledge of the morphological characteristics of predator and prey species. Contrastingly, identifying prey remains or pellets at nest sites is a laborious process, often requiring detailed microscopy work in conjunction with extensive reference collections (e.g. bones and feathers) [21,45]. Our approach provides a cost- and time-efficient method to study raptor diet demographically [21], geographically [36] and now temporally.

## Data Availability

Data and relevant code for this research work are stored in the Dryad Digital Repository: https://doi.org/10.5061/dryad.37pvmcvpj [62].
